# First nationwide survey of infection prevention and control among healthcare facilities in Japan: impact of the national regulatory system

**DOI:** 10.1186/s13756-022-01175-y

**Published:** 2022-11-09

**Authors:** Hidetoshi Nomoto, Hiroki Saito, Masahiro Ishikane, Yoshiaki Gu, Norio Ohmagari, Didier Pittet, Hiroyuki Kunishima, Benedetta Allegranzi, Masaki Yoshida

**Affiliations:** 1 Disease Control and Prevention Center, National Center for Global Health and Medicine, Tokyo, Japan; 2 Emerging and Re-emerging Infectious Diseases, Graduate School of Medicine, Tohoku University, Miyagi, Japan; 3 Faculty of Medicine, Institute of Global Health, University of Geneva, Geneva, Switzerland; 4 Department of Emergency and Critical Care Medicine, St. Marianna University School of Medicine Yokohama Seibu Hospital, Kanagawa, Japan; 5 AMR Clinical Reference Center, National Center for Global Health and Medicine, Tokyo, Japan; 6 Department of Infectious Diseases, Tokyo Medical and Dental University, Tokyo, Japan; 7 Infection Control Programme, University of Geneva Hospitals and Faculty of Medicine, Geneva, Switzerland; 8 Department of Infectious Diseases, St Marianna University School of Medicine, Kanagawa, Japan; 9 Infection Prevention and Control Hub, Integrated Health Services, World Health Organization, Geneva, Switzerland; 10 Department of Infectious Diseases and Infection Control, The Jikei University School of Medicine, Tokyo, Japan

**Keywords:** Antimicrobial resistance, Infection prevention and control, Healthcare associated infection, World Health Organization global survey

## Abstract

**Background:**

Infection prevention and control (IPC) measures in Japan are facilitated by a financial incentive process at the national level, where facilities are categorized into three groups (Tier 1, Tier 2, or no financial incentive). However, its impact on IPC at the facility level using a validated tool has not been measured.

**Methods:**

A nationwide cross-sectional study was conducted from August 2019 to January 2020 to evaluate the situation of IPC programs in Japan, using the global IPC Assessment Framework (IPCAF) developed by the World Health Organization. Combined with the information on the national financial incentive system, the demographics of facilities and each IPCAF item were descriptively analyzed. IPCAF scores were analyzed according to the facility level of care and the national financial incentive system for IPC facility status, using Dunn-Bonferroni and Mann–Whitney U tests.

**Results:**

Fifty-nine facilities in Japan responded to the IPCAF survey: 34 private facilities (57.6%) and 25 public facilities (42.4%). Of these, 11 (18.6%), 29 (49.2%), and 19 (32.3%) were primary, secondary, and tertiary care facilities, respectively. According to the national financial incentive system for IPC, 45 (76.3%), 11 (18.6%), and three (5.1%) facilities were categorized as Tier 1, Tier 2, and no financial incentive system, respectively. Based on the IPCAF total score, more than half of the facilities were categorized as “Advanced” (n = 31, 55.3%), followed by “Intermediate” (n = 21, 37.5%). The IPCAF total score increased as the facility level of care increased, while no statistically significant difference was identified between the secondary and tertiary care facilities (*p* = 0.79). There was a significant difference between Tier 1 and Tier 2 for all core components and total scores. Core components 5 (multimodal strategies for implementation of IPC interventions) and 6 (monitoring/audit of IPC and feedback) were characteristically low in Japan with a median score of 65.0 (interquartile range 40.0–85.0) and 67.5 (interquartile range 52.5–87.5), respectively.

**Conclusions:**

The national financial incentive system was associated with IPC programs at facility level in Japan. The current financial incentive system does not emphasize the multimodal strategy or cover monitoring/audit, and an additional systematic approach may be required to further promote IPC for more practical healthcare-associated infection prevention.

## Background

Infection prevention and control (IPC) is an essential function of healthcare facilities that contributes to patient safety, quality universal health coverage, and achieving sustainable development goals [1]. Promotion of IPC at the facility level helps control antimicrobial resistance (AMR) as well as (re-) emerging infections [2–4]. However, there is a need for a systematic approach to define and assess IPC programs of healthcare facilities at the national level [5]. Therefore, the World Health Organization (WHO) developed evidence-based guidelines on IPC programs [6, 7] and a relevant assessment tool—the IPC Assessment Framework (IPCAF)—in 2018 [8, 9].

The WHO conducted a global survey using the IPCAF in 2019, when their global hand hygiene campaign marked its tenth anniversary [10, 11]. Japan joined this global survey, mainly led by the AMR Clinical Reference Center (AMRCRC), a national focal lead of AMR for healthcare facilities, in collaboration with academic societies. To our knowledge, this is the first IPC-relevant survey at the national level in Japan.

Historically, the Ministry of Health, Labour and Welfare (MHLW) in Japan helped establish IPC programs at the facility level through various measures, among which the major facilitator was the national regulatory system of healthcare with a financial incentive (FI) to promote IPC. In 1996, healthcare facilities meeting certain IPC standards began to receive an FI (0.6 USD per patient per day). This FI system was paused in early 2000s, however, in 2010, the MHLW in Japan re-introduced an FI system of 10 USD per patient per admission. [12]. In 2012, a regional IPC network with multiple surrounding institutions was incorporated as a condition for the FI system, which was subsequently upgraded. If the regional IPC network was established with a facility as the main IPC “hub” of the network, the facility was further classified into a Tier 1 (40 USD per patient per admission) facility. Small- and medium-sized facilities that met the criteria for the basic FI system but did not assign a full-time IPC manager as required for the Tier 1 facilities were classified as Tier 2 (10 USD per patient per admission) [13]. As such, facility-level IPC programs in Japan are heavily guided by the FI system.

Although the national IPC-related FI system was put in place more than a decade ago, the assessment of IPC at the facility level using a globally validated tool has not been conducted nationwide in Japan. Therefore, we conducted a cross-sectional study to evaluate the situation of IPC programs using WHO global survey data collected from healthcare facilities in Japan. The study objective was to assess the overall characteristics of IPC programs across multiple Japanese health facilities, and the impact of the facility of care and the national IPC-related FI system on them.

## Methods

### Ethics statement

According to the Ministry of Education, Culture, Sports, Science and Technology, and MHLW in Japan [14], ethical review and informed consent were not required because no individual patient-level data were used, and no data could be linked to any individual.

### Study design, setting, and sampling

This nationwide cross-sectional survey in Japan was conducted from August 2019 to January 2020, as part of the WHO Global Survey. Survey participation of healthcare facilities was called through AMRCRC, the Japanese Association for Infectious Diseases, the Japanese Society of Intensive Care Medicine, and the Japanese Society for IPC. They made an announcement of the WHO global survey through multiple channels, such as a post on their website and their social networking service and a group email. Participating facilities responded to an online survey for the IPCAF [15].

### Study participants and data collection

#### Questionnaire survey of IPCAF

The IPCAF comprises 81 questions across eight core components (CCs) of IPC programs identified by the WHO recommendations [8]. It enables staff of healthcare facilities to assess, identify the gap of, and promote IPC programs at the facility level, and can also be used to assess the overall IPC situation at both national and regional levels. The score totals 800 (each component = 100 maximum).

The backgrounds and demographics of the respondents’ facilities were collected. The following data were collected through IPCAF:

CC 1, IPC program;

CC 2, IPC guidelines;

CC 3, IPC education and training;

CC 4, Healthcare-associated infection (HAI) surveillance;

CC 5, multimodal strategies for implementation of IPC interventions;

CC 6, monitoring/audit of IPC and feedback;

CC 7, workload, staffing, and bed occupancy;

CC 8, built environment, materials, and equipment for IPC at the facility level.

Each CC was calculated using a score of 0–100 [8]. Based on the total score, the facilities were classified into four categories: (i) 0–200, “Inadequate” (IPC CCs’ implementation was deficit); (ii) 201–400, “Basic” (some aspects of the IPC components were in place but not sufficiently implemented); (iii) 401–600, “Intermediate” (most aspects of IPC CCs were appropriately implemented); and (iv) 601–800, “Advanced” (IPC CCs were fully implemented), according to the WHO recommendations [8].

#### Definition of facility level of care and the national IPC-related FI system

Facility level of care was collected as part of the WHO global survey, and categorized into three categories:

(i) Primary level healthcare facility, defined as a facility with mainly internal medicine, obstetrics and gynecology, pediatrics, or general surgery with few specialties, which could be referred to as a district or rural hospital with limited laboratory services;
(ii) Secondary level healthcare facility, defined as a facility with more specialties, which could be referred to as regional hospitals with bed sizes typically ranging from 200 to 800 beds;
(iii) Tertiary level healthcare facility, defined as a facility with highly specialized services such as cardiology, intensive care unit, and special imaging unit, which could be referred to as a teaching hospital or national hospital with a bed size typically ranging from 300 to 1500 beds [8].

Information on the facility status of the national IPC-related FI system was further obtained and categorized into three groups: (i) Tier 1, (ii) Tier 2, and (iii) no FI, depending on the requirements of each facility met [13].

### Statistical analyses

We first describe the characteristics of respondents through the global survey in Japan. Second, the IPCAF scores were compared by (i) the facility level of care and (ii) the FI facility status for IPC. Third, we conducted a detailed descriptive analysis of the components in Japan that scored low compared to the same survey results from other developed countries [16, 17]. Continuous variables are shown as median with interquartile range (IQR). To compare the FI facility status for IPC, we conducted the only comparison of facilities with Tier 1 and Tier 2, using Mann–Whitney U test, because of the small number of facilities with no FI for IPC. Significance was defined as *P* < 0.05. The Dunn-Bonferroni correction was performed to compare facilities with different levels of care. All analyses were performed using SPSS Statistics version 27 (IBM Corp., Armonk, NY, USA).

## Results

### Respondents’ characteristics

A total of 59 facilities across 21 prefectures out of 47 prefectures in Japan responded to the IPCAF survey (Table 1). The most frequent respondents of the participating facilities were IPC nurses (n = 38, 64.4%). There were 34 private facilities (57.6%) and 25 public facilities (42.4%). About the facility level of care, there were 11 (18.6%) primary, 29 (49.2%) secondary, and 19 (32.3%) tertiary level health care facilities. Most facilities had their own IPC committees (n = 56, 94.9%). According to the national IPC-related FI system, 45 (76.3%), 11 (18.6%), and 3 (5.1%) facilities were categorized as Tier 1, Tier 2, and no FI, respectively (Table 1). More than half of the facilities had “Advanced” status based on the IPCAF total score (n = 31, 55.3%) followed by those with “Intermediate” status (n = 21, 37.5%) and those with “Basic” status (n = 4, 8.0%) with the remaining three facilities with unknown total score due to incomplete answers.

**Table 1:** Descriptive characteristics of respondents (N = 59)

Variable	Number	Proportion (%)
*Occupation*		
Doctor	20	33.9
Nurse	38	64.4
Pharmacist	1	1.7
*Facility type*		
Private	34	57.6
Public	25	42.4
*Facility level of care*		
Primary	11	18.6
Secondary	29	49.2
Tertiary	19	32.2
*IPC role*		
IPC committee member	56	94.9
IPC focal person	3	5.1
*FI category for IPC*		
Tier 1	45	76.3
Tier 2	11	18.6
No financial incentive	3	5.1
IPCAF IPC level*		
Inadequate (scores: 0–200)	0	0
Basic (scores: 201–400)	4	8.0
Intermediate (scores: 401–600)	21	37.5
Advanced (scores: 601–800)	31	55.3

*IPC* infection prevention and control, *IPCAF* Infection Prevention and Control Assessment Framework, *FI* financial incentive

*Three facilities were excluded from the analysis because of incomplete answers

### IPCAF score stratified by facility level of care

**Fig. 1:**
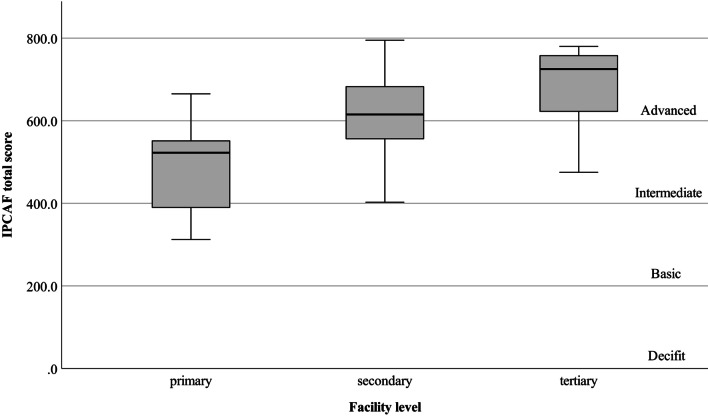
Infection Prevention and Control Assessment Framework (IPCAF) total score, stratified by the facility level of care. The median total scores for all types, and healthcare facilities with tertiary, secondary, and primary level of care were 627.5 (528.7–712.5), 725.0 (617.5–759.3), 615.0 (547.5–687.5), and 522.5 (382.5–570.0), respectively

The median (IQR) total IPCAF score was 627.5 (IQR 528.7–712.5), while for tertiary, secondary, and primary care facilities it was 725.0 (617.5–759.3), 615.0 (547.5–687.5), and 522.5 (382.5–570.0), respectively (Fig. 1, Table 2). The total score increased as the facility level of care increased, although the difference did not reach statistical significance between secondary and tertiary care facilities (*p* = 0.79). CCs 1, 7, and 8, and the total IPCAF scores were significantly higher among secondary than primary care facilities (Table 2). CCs 1 and 2 scores were significantly higher among tertiary than secondary care facilities. All CCs and the total scores were significantly higher among tertiary than primary care facilities.

**Table 2:** Infection Prevention and Control Assessment Framework (IPCAF): comparative analysis of core components by facility level of care (N = 59)

Core components	Facility level of care				*P*-value**		
	All (N = 59)	Tertiary (n = 19, 32.2%)	Secondary (n = 29, 49.2%)	Primary (n = 11,18.6%)	Tertiary vs. Secondary	Tertiary vs. Primary	Secondary vs. Primary
1. IPC programs	82.5 (65.0–90.0)	85.0 (82.5–95.0)	80.0 (68.7–88.7)	60.0 (52.5–75.0)	.040	< .001	.022
2. IPC guidelines	90.0 (80.0–100)	100 (90.0–100)*	87.5 (78.7–100)*	72.5 (62.5–90.0)	.046	< .001	.112
3. IPC education and training	75.0 (60.0–85.0)	85.0 (70.0–95.0)	72.5 (60.0–85.0)*	60.0 (50.0–70.0)	.091	.004	.342
4. HAI surveillance	77.5 (61.8–87.5)	85.0 (77.5–92.5)	77.5 (62.5–82.5)	60.0 (40.0–77.5))	.054	.001	.147
5. Multimodal strategies	65.0 (40.0–85.0)	85.0 (40.0–95.0)	65.0 (50.0–85.0)	45.0 (25.0–60.0)	.673	.017	.154
6. Monitoring/audit of IPC practices and feedback	67.5 (52.5–87.5)	82.5 (60.0–90.0)	67.5 (56.2–85.0)	50.0 (32.5–65.0)	.384	.010	.184
7. Workload, staffing and bed occupancy	85.0 (55.0–100)	95.0 (75.0–100)	85.0 (62.5–100)	50.0 (40.0–85.0)	.682	.003	.039
8. Built environment, materials, and equipment for IPC	97.5 (87.5–97.5)	100 (92.5–100)	97.5 (91.2–100)	87.5 (77.5–90.0)	1.000	.002	.012
Total	627.5 (528.7–712.5)	725.0 (617.5–759.3)	615.0 (547.5–687.5)	522.5 (382.5–570.0)	.079	< .001	.033

Continuous variable data are presented as median (IQR)

*IPC* infection prevention and control, *HAI* healthcare-associated infection

*One facility was excluded from the analysis because of incomplete answers

**The Dunn-Bonferroni correction was performed

### IPCAF scores stratified by the FI facility status for IPC

**Fig. 2:**
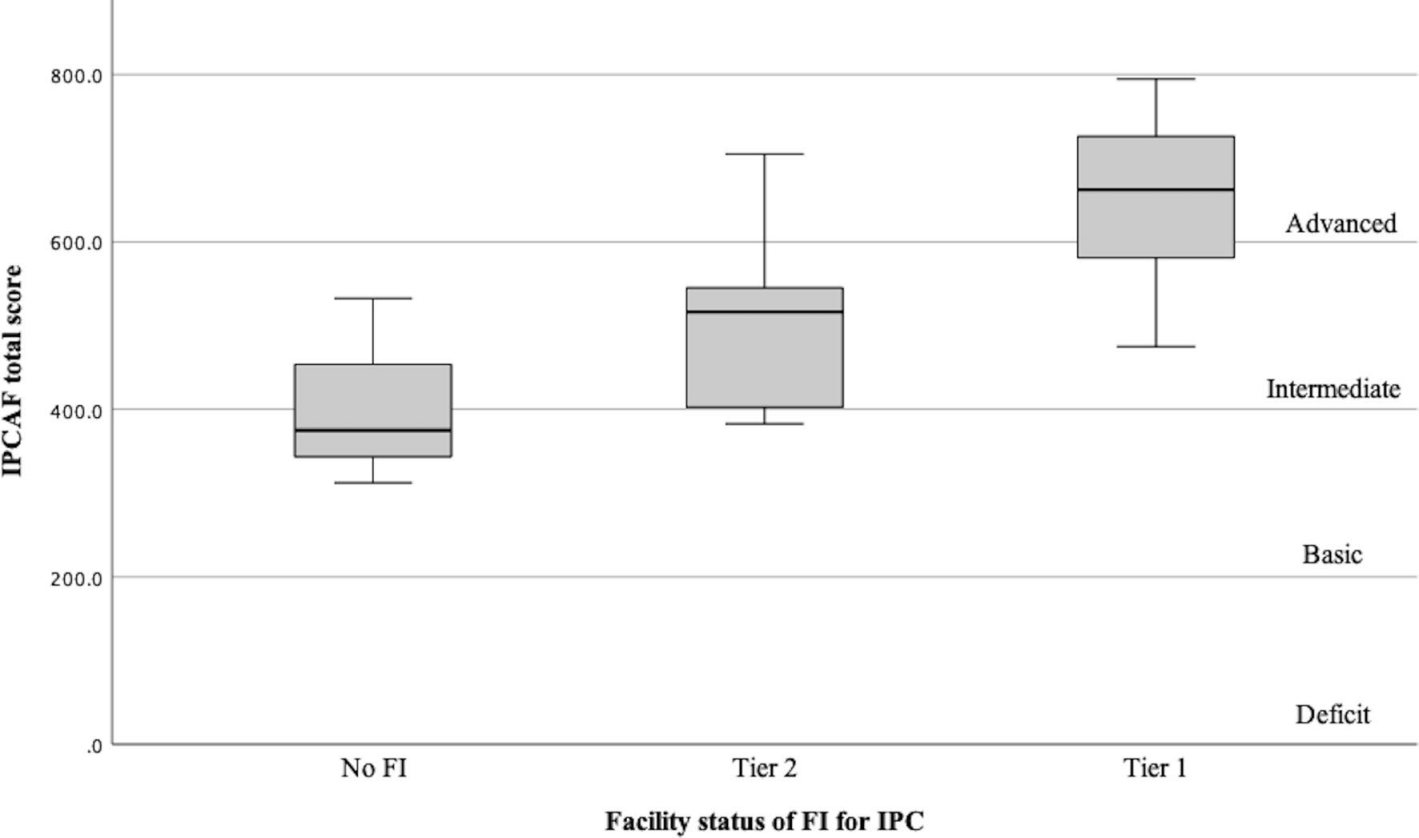
Infection Prevention and Control Assessment Framework (IPCAF) total score, stratified by the facility status of the national financial incentive system for infection prevention and control

The total IPCAF score and each CC score were also compared across the categories of the national IPC-related FI system (Fig. 2, Table 3). The median (IQR) total scores of facilities with Tier 1, Tier 2, and no FI were 662.5 (575.0–735.0), 516.2 (401.2–570.6), and 375.0 (343.8–453.8), respectively. Tier 1 facilities scored significantly higher for the total IPCAF score and all CCs than Tier 2 facilities.

**Table 3:** Infection Prevention and Control Assessment Framework (IPCAF): comparative analysis of core components by the facility status of the national financial incentive system for infection prevention and control (N = 59)

Core components	Facility FI status for IPC			P-value**
	Tier 1 (n = 45, 76.3%)	Tier 2 (n = 11, 18.6%)	No FI* (n = 3, 5.1%)	Tier 1 vs. Tier 2
1. IPC programs	85.0 (77.5–95.0)	60.0 (55.0–70.0)	35.0	< .001
2. IPC guidelines	92.5 (82.5–100)^†^	67.5 (57.5–90.0)	72.5	.001
3. IPC education and training	80.0 (65.0–87.5)	60.0 (50.0–77.5) ^†^	60.0	.014
4. HAI surveillance	82.5 (72.5–89.3) ^†^	60.0 (40.0–70.0)	40.0	< .001
5. Multimodal strategies	75.0 (52.5–90.0)	45.0 (35.0–55.0)	5.0	.017
6. Monitoring/audit of IPC practices and feedback	75.0 (57.5–90.0)	60.0 (40.0–77.5)	32.5	.028
7. Workload, staffing and bed occupancy	90.0 (75.0–100)	50.0 (45.0–85.0)	40.0	.011
8. Built environment, materials, and equipment for IPC	100 (92.5–100)	87.5 (77.5–95.0)	90.0	.001
Total	662.5 (575.0–735.0)	516.2 (401.2–570.6)	375.0 (343.8–453.8)	.001

Continuous variable data are presented as median (IQR)

Comparison between Tier 1, Tier 2, and no FI was performed because of the small sample size of no FI

*IPC* infection prevention and control, *HAI* healthcare-associated infection, *FI* financial incentive

*IQR was not described because of the small sample size (n = 3)

**Mann–Whitney U test was performed

^†^One facility was excluded from the analysis because of incomplete answers

### Core components 5 and 6 in Japan

Compared to other CCs, CCs 5 (multimodal strategies for implementation of IPC interventions) and 6 (monitoring/audit of IPC and feedback) were low in Japan (65.0 [40.0–85.0] and 67.5 [52.5–87.5], respectively) (Table 4, 5). For CC 5, 40.7% of the facilities reported they did not use multimodal strategies to implement IPC interventions. Elements such as education and training, and monitoring and feedback were conducted in 42.4% and 52.5% of facilities, respectively, while safety climate and culture change were focused only in 15.3% of facilities. For CC 6, most facilities had a person responsible for monitoring and auditing IPC practices (91.5%); however, only two-thirds of the facilities (69.5%) had a monitoring plan in place. Hand hygiene compliance (81.4%) and consumption of alcohol-based hand rub or soap (88.2%) and antimicrobial agents (93.3%) were commonly monitored. Conversely, intravascular catheter insertion care (50.9%), wound dressing change (27.2%), cleaning of the ward environment (49.2%), and disinfection and sterilization of medical equipment or instruments (52.6%) were less frequently monitored. In addition, 35.6% of healthcare facilities did not measure the WHO Hand Hygiene Self-Assessment Framework despite the above-mentioned large proportion of facilities monitoring hand hygiene compliance. Only one-third of the facilities (32.3%) used a scale to assess safety culture parameters.

**Table 4:** Detailed results of Infection Prevention and Control Assessment Framework (IPCAF): core component 5 (N = 59)

Element	Answer	Score	Number	Proportion (%)
Using multimodal strategies to implement IPC interventions	No	0	24	40.7
	Yes	15	35	59.3
*Multimodal strategies including any or all of the following elements:*				
Choose one answer (the most accurate) per element				
System change	Element not included	0	17	28.8
	Interventions to ensure the necessary infrastructure and continuous availability of supplies are in place	5	27	45.8
	Interventions to ensure the necessary infrastructure and continuous availability of supplies are in place and addressing ergonomics and accessibility, such as the best placement of central venous catheter set and tray	10	15	25.4
Education and training	Element not included	0	13	22.0
	Written information and/or oral instruction and/or e-learning only	5	21	35.6
	Additional interactive training sessions (includes simulation and/or bedside training)	10	25	42.4
Monitoring and feedback	Element not included	0	11	18.6
	Monitoring compliance with process or outcome indicators	5	17	28.8
	Monitoring compliance and providing timely feedback of monitoring results to health care workers and key players	10	31	52.5
Communications and reminders	Element not included	0	11	18.6
	Reminders, posters, or other advocacy/awareness-raising tools to promote the intervention	5	32	54.2
	Additional methods/initiatives to improve team communication across units and disciplines	10	16	27.1
Safety climate and culture change	Element not included	0	22	37.3
	Managers/leaders show visible support and act as champions and role models, promoting an adaptive approach and strengthening a culture that supports IPC, patient safety and quality	5	28	47.5
	Additionally, teams and individuals are empowered so that they perceive ownership of the intervention	10	9	15.3
A multidisciplinary team used to implement IPC multimodal strategies	Not exist	0	9	15.3
	Exist	15	50	84.7
Regular link to colleagues from quality improvement and patient safety to develop and promote IPC multimodal strategies	Not exist	0	13	22.0
	Exist	10	46	78.0
Strategies including bundles or checklists	Not exist	0	21	35.6
	Exist	10	38	64.4

*PC* infection prevention and control

IPC core component 5 refers to multimodal strategies for implementation of IPC interventions

**Table 5:** Detailed results of Infection Prevention and Control Assessment Framework (IPCAF): core component 6 (N = 59)

Element	Answer	Score	Number	Proportion (%)
Trained personnel responsible for monitoring/audit of IPC practices and feedback	Do not exist	0	5	8.5
	Exist	10	54	91.6
A well-defined monitoring plan with clear goals, targets, and activities	Not conducted	0	18	30.6
	Conducted	7.5	41	69.5
Processes and indicators monitored in your facility (check all that apply)	None	0	1	1.7
	Hand hygiene compliance (using the WHO hand hygiene observation tool or equivalent)	5	48	81.4
	Intravascular catheter insertion and/or care	5	30	50.9
	Wound dressing change	5	16	27.2
	Transmission-based precautions and isolation to prevent the spread of multidrug resistant organisms	5	39	66.2
	Cleaning of the ward environment	5	29	49.2
	Disinfection and sterilization of medical equipment/instrument	5	31	52.6
	Consumption/usage of alcohol-based hand rub or soap	5	52	88.2
	Consumption/usage of antimicrobial agents	5	55	93.3
	Waste management	5	32	54.3
Frequency of the WHO Hand Hygiene Self-Assessment Framework Survey undertaken	Never	0	21	35.6
	Periodically, but no regular schedule	2.5	21	35.6
	At least annually	5	17	28.9
Feedbacking auditing reports on the state of the IPC activities/performance	No reporting	0	2	3.4
	Within the IPC team	2.5	48	81.4
	To department leaders and managers in the areas being audited	2.5	39	66.2
	To frontline healthcare workers	2.5	45	76.3
	To the IPC committee or quality of care committees or equivalent	2.5	48	81.4
	To hospital management and senior administration	2.5	33	56
The regular reporting of monitoring data (at least annually)	Not conducted	0	5	8.5
	Conducted	10	54	91.6
Monitoring and feedback of IPC processes and indicators performed in a “blame-free” institutional culture aimed at improvement and behavioral change	Not conducted	0	15	25.5
	Conducted	5	44	74.6
Assessing safety cultural factors in your facility (for example, by using other surveys such as HSOPSC, SAQ, PSCHO, HSC)	Not conducted	0	40	67.8
	Conducted	5	19	32.3

*IPC* infection prevention and control, *WHO* World Health Organization, *HSOPSC* Hospital Survey on Patient Safety Culture, *SAQ* Safety attitudes questionnaire, *PSCHO* Patient safety climate in healthcare organizations, *HSC* Hospital Safety Climate Scale

IPC core component 6 refers to monitoring/audit and feedback of IPC practices

## Discussion

IPC at the health facility level across Japan was evaluated for the first time through a globally validated tool, IPCAF, and the level of IPC programs was “Advanced” at more than half of the facilities. We further stratified facilities by FI facility status for IPC, a unique factor regulated by the Japanese healthcare policy. Facilities with a higher level of care and a Tier 1 FI status, whose IPC requirements were more comprehensive, scored higher in IPCAF, suggesting that their IPC programs were better resourced and implemented.

The IPCAF is a globally validated tool that highlights IPC characteristics across countries. The median total IPCAF score in Japan was 627.5, which was categorized as “Advanced” IPC level. The global survey conducted in 2019 showed the median score was 632.5 (IQR 482.5–710) for upper-middle-income countries and 657.5 (IQR 562.5–717.5) for high-income countries [10]. Japan is categorized as a high-income country and its score in our study also corresponds to these areas [18]. However, since the composition of facility level of care in the global survey is not uniform to that of our study, we need to be cautious about the simple comparison of these results.

On the other hand, in a study of 736 acute care hospitals in Germany, the median score was 690 (640–762.5) [16]. A similar study was conducted in Austria with a median score of 620 (567.5–709) [17]. Both countries were also ranked as “Advanced” in the IPCAF, suggesting well-developed IPC programs.

Compared to these study results, Japan scored higher on CC 7 (workload, staffing, and bed occupancy) and CC 8 (built environment, materials, and equipment for IPC). These CCs correspond to the hardware part of the healthcare facilities. Therefore, resource investment in such aspects of IPC seems appropriate in Japan.

IPCAF total scores tended to be higher for facilities with higher level of care and facilities receiving higher financial incentives. These two factors are related to each other and cannot be considered in isolation, as institutions with high level of care tend to gain higher financial incentives for IPC. Nonetheless, such allocation of dedicated resources for IPC may contribute to improvement of the national IPC level for better quality of care [10]. Healthcare delivery, including IPC measures at the facility level in Japan, has been historically guided by the payment system for healthcare; in particular, FI per patient admission is paid to a hospital if it meets certain IPC requirements set by the government, a unique system compared to other countries. Facilities that receive FI are organized in accordance with the stipulated IPC programs set out by the government. This national IPC-related FI system in Japan mainly defines members of an IPC team, including designated IPC experts, and sets IPC-related training and facility-level guideline development. These factors required by FI system could contribute to IPC CCs measured in IPCAF. In addition, the concept of “regional collaboration with multiple institutions on IPC” was introduced in the 2012 revision [13]. Since then, Tier 1 facilities have been required to form a regional support network with the surrounding Tier 2 facilities. This might have further promoted exchange of the information on and experience in IPC measures, not only in terms of the dissemination of knowledge on IPC measures, but also mutual evaluation of standardized IPC measures among participating facilities.

Here, we focus on particular CCs: our analysis showed that CCs5 and 6 had a characteristically lower score in Japan than in Austria, Germany and other high-income countries [10, 16, 17]. Multimodal strategy, CC 5, is an implementation strategy to improve a target outcome or to change behavior, comprising several elements or components implemented in an integrated way [7]. It has been shown to be effective to improve IPC practices, and should ideally play a major role on IPC programs. Adaptation of the multimodal strategy at both national and facility level will further facilitate IPC programs in Japan. Also, monitoring and auditing of IPC practices, CC 6, is strongly associated with CC 5 as monitoring and auditing ensure adherence to IPC practices promoted by the multimodal strategy. While most facilities (91.5%) had a person in charge of monitoring and auditing IPC practices, substantially fewer facilities (69.5%) had a monitoring plan in place. In addition, while consumable items such as disinfectants and antimicrobials were frequently monitored, items or processes more directly related to patient safety, such as intravascular catheter insertion/care, wound dressing change, cleaning of the ward environment, disinfection, and sterilization of medical equipment and instruments, were less frequently monitored. Such signs of inadequate safety culture for HAI prevention and the lack of granularity of IPC-related monitoring may arise from insufficient utilization of the established HAI surveillance system as an outcome measure in Japan. The overall picture of the disease burden of HAIs in Japan is not clear, partly because the national FI system for IPC does not mandate reporting of outcome measures for HAIs. In a multicenter cross-sectional study, Sakamoto and colleagues revealed that the degree of compliance with evidence-based HAIs preventive measures was determined by each hospital’s resources and organizational attitude toward patient safety [19]; while the proportion of facilities conducting surveillance for CLABSI, CAUTI, and ventilator-associated pneumonia were 55.9%, 34.9%, and 31.4%, respectively, in 2012, the situation remained almost unchanged in their following evaluation in 2016 [20]. To further promote practical IPC programs at the facility level, it is worth considering a system that fully utilizes outcome-oriented surveillance, where HAI rates are continuously measured as an indicator [21]. Strategic planning and implementation of IPC measures based on results from an active surveillance system will likely contribute to a more effective risk reduction of HAIs in Japan.

We recognize study limitations. First, we used both the original English version of the IPCAF tool and the one translated into Japanese for the survey [15] (see Acknowledgements). Because of the translation, there might be some items that do not fully convey the original intent of the tool, despite the fact that several Japanese experts fluent in English were involved in the translation. Next, although the IPCAF tool is carefully designed as a self-assessment instrument, some questions require an understanding of the WHO methodology, which some Japanese respondents might not be accustomed to. In addition, IPCAF is a self-reported tool and thus, responses could have also been susceptible to a certain degree of social desirability bias, whereby respondents prefer to select the best answer over the true answer. Finally, because of the relatively small sample size, the results may not reflect the IPC situation in all health facilities in Japan. However, the Japanese Society for IPC—the main channel used for survey participation—expectedly reached out to most IPC practitioners in Japan. This also inhibited us from performing logistic regression analyses to adjust for potential confounders such as facility type, and we could not assess whether the facility status of the national IPC-related FI system was independently associated with IPC programs in Japan. Nevertheless, we believe that this first-ever evaluation of facility IPC programs using a globally validated tool provides important insight into the current situation of IPC of facilities in Japan and helps identify issues for future improvement in IPC implementation.

## Conclusions

We conducted a nationwide survey in Japan to evaluate the facility-level IPC programs through a globally validated tool. Our study is conducted for the first time in Asian countries according to the literature. The facility level of care and the FI facility status for IPC may be associated with IPC programs in Japan. The current FI system does not cover monitoring/audit, and focusing on multimodal strategy of IPC implementation and outcome measures of the IPC implementation may further strengthen the IPC programs at facility level in Japan.

## Data Availability

The datasets used and/or analyzed during the current study are available from the corresponding author upon reasonable request.

## Abbreviations

AMRCRC: Antimicrobial Resistance Clinical Reference Center
AMR: Antimicrobial resistance
CAUTI: Catheter-associated urinary tract infection
CC: Core components
CLABSI: Central line-associated bloodstream infection
FI: Financial incentive
HAI: Healthcare-associated infection
IPC: Infection prevention and control
IPCAF: Infection Prevention and Control Assessment Framework
IQR: Interquartile range
MHLW: Ministry of Health, Labour and Welfare
WHO: World Health Organization

## References

[1] Storr J, Kilpatrick C, Allegranzi B, Syed SB (2016). Redefining infection prevention and control in the new era of quality universal health coverage. J Res Nurs.

[2] Baur D, Gladstone BP, Burkert F, Carrara E, Foschi F, Döbele S, Tacconelli E (2017). Effect of antibiotic stewardship on the incidence of infection and colonisation with antibiotic-resistant bacteria and *Clostridium difficile* infection: a systematic review and meta-analysis. Lancet Infect Dis.

[3] Rhee C, Baker M, Vaidya V, Tucker R, Resnick A, Morris CA, Klompas M (2020). CDC Prevention Epicenters Program. Incidence of nosocomial COVID-19 in patients hospitalized at a large US academic medical center. JAMA Netw Open.

[4] Ohmagari N (2019). National Action Plan on Antimicrobial Resistance (AMR) 2016–2020 and relevant activities in Japan. Glob Health Med..

[5] Zingg W, Storr J, Park BJ, Ahmad R, Tarrant C, Castro-Sanchez E, Tomczyk S, Kilpatrick C, Allegranzi B, Cardo D, Pittet D (2019). 2017 Geneva IPC-Think Tank. Implementation research for the prevention of antimicrobial resistance and healthcare-associated infections; 2017 Geneva infection prevention and control (IPC)-think tank (part 1). Antimicrob Resist Infect Control.

[6] Zingg W, Holmes A, Dettenkofer M, Goetting T, Secci F, Clack L, Allegranzi B, Magiorakos AP, Pittet D (2015). Systematic review and evidence-based guidance on organization of hospital infection control programmes (SIGHT) study group. Hospital organisation, management, and structure for prevention of health-care-associated infection: a systematic review and expert consensus. Lancet Infect Dis.

[7] World Health Organization. Guidelines on core components of infection prevention and control programmes at the national and acute health care facility level, 2016. http://apps.who.int/iris/bitstream/10665/251730/1/9789241549929-eng.pdf?ua=1. Accessed 21 Sept 2021. 27977095

[8] World Health Organization. Infection prevention and control assessment and control assessment framework at the facility level. https://www.who.int/infection-prevention/tools/core-components/IPCAF-facility.PDF?ua=1. Accessed 21 Sept 2021.

[9] Tomczyk S, Aghdassi S, Storr J, Hansen S, Stewardson AJ, Bischoff P, Gastmeier P, Allegranzi B (2020). Testing of the WHO Infection Prevention and Control Assessment Framework at acute healthcare facility level. J Hosp Infect.

[10] Tomczyk S, Twyman A, de Kraker MEA, Coutinho Rehse AP, Tartari E, Toledo JP, Cassini A, Pittet D, Allegranzi B (2022). The first WHO global survey on infection prevention and control in health-care facilities. Lancet Infect Dis.

[11] Peters A, Borzykowski T, Tartari E, Kilpatrick C, Mai HCS, Allegranzi B, Pittet D (2019). “Clean care for all - It's in your hands”: The May 5th, 2019 World Health Organization SAVE LIVES: Clean Your Hands campaign. Int J Infect Dis.

[12] Morikane K (2012). Infection control in healthcare settings in Japan. J Epidemiol.

[13] Morikane K (2014). Local partnership in infection control. Nippon Naika Gakkai Zasshi.

[14] Ministry of Education, Culture, Sports, Science and Technology, Ministry of Health, Labour and Welfare. Ethical guidelines for medical research involving human subjects (provisional translation). https://www.mhlw.go.jp/file/06-Seisakujouhou-10600000-Daijinkanboukouseikagakuka/0000080278.pdf. Accessed 21 Sept 2021.

[15] World Health Organization. Infection prevention and control assessment and control assessment framework at the facility level (Japanese version). https://www.who.int/nepal/activities/supporting-elimination-of-kala-azar-as-a-public-health-problem/docs/default-source/integrated-health-services-(ihs)/infection-prevention-and-control/hand-hygiene/tools/japan/ipcaf-japanese. Accessed 21 Sept 2021.

[16] Aghdassi SJS, Hansen S, Bischoff P, Behnke M, Gastmeier P (2019). A national survey on the implementation of key infection prevention and control structures in German hospitals: results from 736 hospitals conducting the WHO Infection Prevention and Control Assessment Framework (IPCAF). Antimicrob Resist Infect Control.

[17] Aghdassi SJS, Grisold A, Wechsler-Fördös A, Hansen S, Bischoff P, Behnke M, Gastmeier P (2020). Evaluating infection prevention and control programs in Austrian acute care hospitals using the WHO Infection Prevention and Control Assessment Framework. Antimicrob Resist Infect Control.

[18] Tartari E, Tomczyk S, Pires D, Zayed B, Coutinho Rehse AP, Kariyo P, Stempliuk V, Zingg W, Pittet D, Allegranzi B (2021). Implementation of the infection prevention and control core components at the national level: a global situational analysis. J Hosp Infect.

[19] Sakamoto F, Sakihama T, Saint S, Greene MT, Ratz D, Tokuda Y (2014). Health care-associated infection prevention in Japan: the role of safety culture. Am J Infect Control.

[20] Sakamoto F, Asano K, Sakihama T, Saint S, Greene MT, Patel P, Ratz D, Tokuda Y (2019). Changes in health care-associated infection prevention practices in Japan: results from 2 national surveys. Am J Infect Control.

[21] Sakamoto F (2017). Healthcare-associated infection surveillance as a strategy against antimicrobial-resistance: significance and future directions. Jpn J Qual Safe Healthc.

